# Single-Step Genome Wide Association Study Identifies QTL Signals for Untrimmed and Trimmed Thigh Weight in Italian Crossbred Pigs for Dry-Cured Ham Production

**DOI:** 10.3390/ani11061612

**Published:** 2021-05-29

**Authors:** Valentino Palombo, Mariasilvia D’Andrea, Danilo Licastro, Simeone Dal Monego, Sandy Sgorlon, Misa Sandri, Bruno Stefanon

**Affiliations:** 1 Dipartimento Agricoltura, Ambiente e Alimenti, Università degli Studi del Molise, Via de Sanctis Snc, 86100 Campobasso, Italy; abg@unimol.it; 2 ARGO Open Lab Platform for Genome Sequencing, AREA Science Park, Padriciano, 99, 34149 Trieste, Italy; danilo.licastro@areasciencepark.it (D.L.); simeone.dalmonego@areasciencepark.it (S.D.M.); 3 Dipartimento di Scienze Agroambientali, Alimentari e Animali, Università di Udine, Via Delle Scienze, 208, 33100 Udine, Italy; sandy.sgorlon@uniud.it (S.S.); misa.sandri@uniud.it (M.S.); bruno.stefanon@uniud.it (B.S.)

**Keywords:** dry-cured ham, ssGWAS, pig, protected designation of origin, thigh weight

## Abstract

**Simple Summary:**

Along with the traditional traits, swine breeding programs for Italian dry-cured ham production have recently aimed to include novel phenotypes. The identification of the genomic regions underlying such new traits helps to untangle their genetic architecture and may provide useful information to be integrated in genetic selection. With this aim, we estimated genetic parameters and conducted a single step genome wide association studies (GWAS) on untrimmed and trimmed thigh weight considering two pig crossbred lines approved for Italian Protected Designation of Origin ham production. Quantitative trait loci (QTLs) were characterized based on the variance of 10-SNP sliding windows genomic estimated breeding values. In particular, we identified interesting QTL signals on several chromosomes, notably on chromosome 4, 6, 7 and 15. A high heritability and genetic correlation were observed for the two traits under investigation and although independent studies including other pig populations are required to disentangle the possible effects of specific linkage disequilibrium in our population, our findings suggest that such QTL could be investigated in future pig breeding programs to improve the reliability of genomic estimated breeding values for the dry-cured ham production.

**Abstract:**

Protected Designation of Origin (PDO) dry-cured ham is the most important product in the Italian pig breeding industry, mainly oriented to produce heavy pig carcasses to obtain hams of the right weight and maturity. Recently, along with the traditional traits swine breeding programs have aimed to include novel carcass traits. The identification at the genome level of quantitative trait loci (QTLs) affecting such new traits helps to reveal their genetic determinism and may provide information to be integrated in prediction models in order to improve prediction accuracy as well as to identify candidate genes underlying such traits. This study aimed to estimate genetic parameters and perform a single step genome wide association studies (ssGWAS) on novel carcass traits such as untrimmed (UTW) and trimmed thigh weight (TTW) in two pig crossbred lines approved for the ham production of the Italian PDO. With this purpose, phenotypes were collected from ~1800 animals and 240 pigs were genotyped with Illumina PorcineSNP60 Beadchip. The single-step genomic BLUP procedure was used for the heritability estimation and to implement the ssGWAS. QTL were characterized based on the variance of 10-SNP sliding window genomic estimated breeding values. Moderate heritabilities were detected and QTL signals were identified on chromosome 1, 4, 6, 7, 11 and 15 for both traits. As expected, the genetic correlation among the two traits was very high (~0.99). The QTL regions encompassed a total of 249 unique candidate genes, some of which were already reported in association with growth, carcass or ham weight traits in pigs. Although independent studies are required to further verify our findings and disentangle the possible effects of specific linkage disequilibrium in our population, our results support the potential use of such new QTL information in future breeding programs to improve the reliability of genomic prediction.

## 1. Introduction

The production of Protected Designation of Origin (PDO) dry-cured ham is the most important product in the Italian pig breeding industry with remarkable economic benefits for producers [1]. Parma and San Daniele are the two main Italian dry-cured ham consortia, representing ~50% of the entire Italian production, reaching a total economic value of more than one billion € per year (Qualivita-ISMEA, 2019) [2]. In light of this, the Italian PDO pig breeding industry is manly oriented to produce heavy pig carcasses to obtain hams of the right weight and maturity for PDO markets [3]. Indeed, the relevance of ham weight at slaughtering is an important aspect to guarantee high quality processed products [3]. The control of time and processing conditions during the seasoning period guarantees a high standard level of final product that does not contain additives and preservatives, thus the quality of the fresh legs is of fundamental importance. In this context, the role of the breeding programs able to produce animals with the requested intrinsic characteristics of the meat and legs remains pivotal and must meet strict PDO standards and protocols. Conventionally, Consortia for the protection of Parma and San Daniele admit some purebred subjects, or hybrids obtained from some breeds: traditionally Large White, Landrace, Duroc [3,4], and from crossbreeds derived from them [5,6]. Furthermore, within the circuit of the PDO, pigs are slaughtered with a live weight of ~160 kg (reached at an age at least of 9 months) to obtain hams of 12–14 kg [1]. In general, the ham is the major piece of the pork carcass and thus, changes in ham weight influences significantly the overall carcass value. This is particularly true in Italian PDO markets, where the value of raw hams is nearly 30% of the total carcass market price [7]. In this context, along with the traditional growth and carcass traits [4,8], the genetic selection for new phenotypes for dry-cured ham production is gaining importance [9,10]. Among others, the untrimmed (UTW) and trimmed (TTW) ham weights, not commonly included in selection indexes [9], are now of particular interest in dry-cured ham production. These traits, in combination with other factors, notably backfat thickness, are related to seasoning weight loss [3]. Indeed, reducing the ham weight loss is a pivotal objective in dry-cured ham production, since large weight losses during dry-curing have significant implications on marketable end-product, not only in terms of achievable revenue but also because of their influence on quality [7]. In this regard, it is worth to note that weight loss at the end of seasoning period (at least 12–13 months) represents, by its nature, a troublesome phenotype with a series of traceability problems during dry-curing steps. This clearly has an impact on effectiveness of phenotypic recording systems, especially in terms of time with penalizing effects on the length of the generation interval in selective breeding [7]. For this reason, weight loss at seven days has been recently considered as a valuable alternative trait of interest [1] and is already used by Italian Herdbook in its pure breed selection programs, along with other traditional traits such as common performance, carcass, meat quality, and backfat thickness [11]. Nevertheless, the availability of facilities for ham processing and traceability of individual hams at the processing plant is a prerequisite also for such a novel phenotype [1] and not always easily satisfied. Furthermore, considering its high correlation with other important traits such as lean cuts weight [3], the breeding selection objective is mainly oriented to maintain it constant. In this general context, the use of a more feasible-to-recode and routinely measured phenotypes, such as UTW and TTW, may be helpful although the impact of different trimming processes, which endows the ham with its typical shape, following the salting phase need to be considered. In the light of this, it is important to highlight that when thigh weight increases, evident effects on both reduction of seasoning loss and organoleptic quality of hams have been traditionally described [3]. Ham weight represents one of the main factors that can influence the aptitude of the ham to adsorb salt [10]. Notably, TTW might be of particular interest since many meat defects become visible several hours after death and are particularly noticeable during ham trimming [12].

In the present study, leg traits were collected from the entire population of two pig crossbred lines approved for the ham production of the Italian San Daniele PDO circuit. Although many studies have been focused on purebreds, pig industries favor crossbreeds and few association studies exist [13,14,15,16]. From the entire population, a subset of animals were randomly chosen and genotyped using the Illumina PorcineSNP60 Beadchip [17]. The single-step GWAS (ssGWAS) procedure was performed in order to combine all information available from genotyped and un-genotyped animals and increase the power of association analysis [18]. Moreover, genetic parameters were estimated under the framework of single-step genomic BLUP (ssGBLUP) approach [19].

## 2. Materials and Methods 

### 2.1. Animal and Trait Measurements

The study did not imply modification of the farm and slaughter protocols and data were provided by the farmers and abattoir personnel. Samples were collected from the green and trimmed thigh with the permission of farmers. Animal care and slaughter of the pigs were under the supervision of the veterinarians of the National Health Service. The study followed the guidelines of the Animal Care Committee of the Department of Animal Science of the University of Udine (Italy).

For the study, 1810 pigs reared in five Italian commercial farms, all within the circuit of the PDO for the productions of San Daniele ham, were used. Initially, a group of 230 Large White farrows were introduced in the farms and were mated with Duroc and Goland C21 boars. Large White and Duroc were enrolled in the Italian Registry of the Italian Pig Breeder National Association (Associazione Nazionale Allevatori Suini, ANAS, http://www.anas.it, accessed on 18 February 2021) and the Goland C21 is a commercial line approved for the ham production of the PDO (Gorzagri s.s., Italy, https://goland.it, accessed on 18 February 2021). The litters of Duroc × Large White and Goland C21 × Large White were born from January 2013 to December 2014 and, according to the prescription of the PDO, were reared in the farms for 9 months. The farms had implemented a traceability system, through the application at the birth of a radio-frequency identification (RFID) object tags in the thighs, allowing to individually track pigs in the farm and the slaughter and processing phases. The final live weight of pigs ranged from 145 to 170 kg and during the whole period pigs were housed in groups of 15 animals in a pen with access to an external paddock. Starting from 35 days of age pigs were fed a post-weaning commercial supplement. At around the age of 80 days, pigs were fed diets formulated by the Breeding Association of the Friuli Venezia Giulia (FVG) Region, in compliance with the PDO prescription. In particular, diets were offered to a semi-libitum and formulated for an initial phase of growth (from 80 to 115 days), an intermediated phase of growth (from 115 to 195 days), and for the finishing phase (from 195 to 270 days). Ingredients and chemical composition of the diets are reported in Supplementary Table S1 [20]. 

Pigs were delivered to abattoirs 12 h before slaughter. After slaughter, backfat thickness, and loin thickness were automatically measured and recorded employing Fat-O-Meat’er instrumentation (FOM-Crometec Gmbh, Lünen, Germany), inserting the probe between the third and fourth last rib on the left hot carcass at 8 and 10 cm off the dorsal midline. The carcass lean percentage was calculated and cold carcass weight was recorded for each selected pig, as by the Council Regulation (EC) n. 1234/2007, Annex V, Part B. Backfat thickness was used to grade the carcass in the EUROP grid. Only the U, R, and O carcasses were considered suitable according to PDO production. Moreover, the weight of the right thigh was recorded before and after trimming, which was performed 24 h after slaughter. Trimming consisted of removing part of the fat and the rind to obtain the typical round ham shape. The RFID tracking system was used to collect these data from the animals and guarantee traceability information during the whole pig slaughter line and 1810 untrimmed thigh (UTW) and 1202 trimmed thigh (TTW) weight values were obtained. 

### 2.2. Genotyping and Quality Control

Among the phenotyped animals, 240 pigs [Duroc × Large White (DL) and Goland C21 × Large White (GL)] were randomly chosen for genotyping. The DNA was extracted from the tissue samples using the ExgeneTM Clinic SV commercial kit (Gentaur, Italy). After extraction, the quality and quantity of nucleic acid were assessed by electrophoresis and spectrophotometry with the Nanodrop instrument. The DNA was stored at −20 °C. Four hundred nanograms of genomic DNA were used for marker analysis with the PorcineSNP60 Genotyping BeadChip [17] following the Illumina Infinium HD protocol (San Diego, CA, USA). BeadChips were scanned by HiScan (Illumina) and genotyping data were extracted using Illumina Genome Studio Software using Plink Plugin. This array includes more than 64,232 SNPs, of these 47,446 SNPs were successfully and uniquely mapped on the Sscrofa11.1 genome version (GCA_000003025.6, Ensembl database) excluding sexual chromosome [21]. Thus genotyping data were filtered retaining samples and SNPs with call rates >0.95, minimum allele frequency (MAF) >0.05 and that did not deviate from Hardy-Weinberg equilibrium (considering a *p* > 0.00001). All quality control procedures were performed using the PLINK v.1.07 toolset [9]. After the filtering step, the final data set consisted of 36,569 SNPs in 236 animals. The SNPs provided uniform genome-wide coverage with an average spacing of 60.82 kb (Table S2).

### 2.3. Estimation of Genetic Parameters

The model for statistical trait analysis included the fixed effects of crossbreed, sex, contemporary group (concatenation of birth year and month), age at slaughtering, farm, and abattoir. Variance components were estimated using the Average Information REML method implemented in the AIREMLF90 module from the BLUPF90 family of programs [22]. A single trait mixed linear model was implemented considering the ssGBLUP relationship matrix [23], as follows:

*y* = ***X**b* + ***Z**a* + *e*

where *y* is the vector of investigated traits; ***X*** is the incidence matrix linking records to fixed effects and *b* is the related vector including crossbred (2 levels), sex (2 levels), contemporary group (12 levels), farm (5 levels) and abattoir (5 levels), slaughter age as covariate; ***Z*** is the incidence matrix for random genetic effects, relating records to animals, and *a* is the vector of the individual additive genetic values (computed according to the blended genomic and pedigree relationship ***H*** matrix, described below); and *e* is the vector of random residuals distributed as $\sim N\left(0,I\sigma_{e}^{2}\right)$, where $\sigma_{e}^{2}$ is the residual variance and ***I*** is an identity matrix. The additive genetic effect was modelled according to the (co)variance structures in the single-step framework (ssGBLUP), which is the blended genomic and pedigree relationship matrix (***H***) according to Aguilar et al. [24]. The regular ssGBLUP model [23,25] uses the ***H*** matrix that combines the marker-based (***G***) and pedigree-based (***A***) relationship matrices to replace the numerator relationship matrix (***A***) in the classical animal model [26]. With this approach, all genotypes, phenotype records and pedigree information were considered in one step simultaneously. The mixed model equations need inversion of ***H*** that can be obtained as follows [24]:

$$H^{-1}=A^{-1}+\left[\begin{array}{c} 0\\ \;0\;G^{-1} \end{array}\begin{array}{c} 0\\ -A_{22\;}^{-1} \end{array}\right]$$

where ***A***_22_ is the sub-matrix of the pedigree relationships among the genotyped animals. ***G*** is the genomic relationship matrix as constructed by VanRaden [27]. According to the reference literature, to avoid singularity ***G*** was blended with 5% of ***A***_22_ and the tuning was performed using the default options in the BLUPF90 family of programs, which adjusts ***G*** to have mean of diagonals and off-diagonals equal to ***A***_22_ [27,28]. The heritability (h^2^) was calculated as:

$$h^{2}=\frac{\sigma_{a}^{2}}{\left(\sigma_{a}^{2}+\sigma_{e}^{2}\right)}$$

### 2.4. Genome-Wide Association Mapping

The ssGBLUP approach was employed for the genome-wide association mapping as well, as described by Wang et al. [29]. Briefly, the GEBV solutions were used to estimate marker effects using the equivalence between GBLUP and SNP-BLUP [29], through an iterative process called weighted ssGBLUP (WssGBLUP). In the first round the GEBV solutions were utilized to estimate marker effects based on a ***G*** matrix weighted by the expected marker variance, assumed to be 1 (i.e., the same weight for all markers) [27]. In successive iterations, marker effects were then recalculated with a similar process but with SNP expected variance in ***G*** replaced by the realized variance obtained in the previous iteration. The reweighting process increased the weight of SNP with large effect and decreased those with small effects. A detailed description of the iterative algorithm is outlined in Wang et al. [29]. In our study, updating SNP weights were continued for only two iterations because of the decreasing accuracy of genomic breeding values in the succeeding iterations (Table S3) and according to other studies [29,30,31]. Accuracy of breeding values animals was estimated as Henderson et al. [26] and Hayes et al. [32]:

$$\mathrm{accuracy}=\sqrt{1-{\mathrm{SEP}}^{2}/\sigma_{a}^{2}}$$

where SEP is the standard error of prediction, derived from the diagonal element of the left-hand side inverse of the mixed model equations [26] and $\sigma_{a}^{2}$ is the additive genetic variance. A fivefold cross validation design was used for the estimation of genomic evaluation accuracy. Briefly, a resampling strategy was applied to create five validation sets encompassing the ~20% of the total population and all remaining individuals (80% of the total population) were used as the training data set. The 2-trait model was used to estimate (co)variance components genetic correlation among the two traits under investigation as:

$$r_{g}=\frac{{\mathrm{cov}}_{g}}{\sqrt{V_{g1}V_{g2}}}$$

where r_g_ is the genetic correlation, cov_g_ is genetic covariance between trait 1 and trait 2, V_g1_ and V_g_*_2_* are the genetic variance of trait 1 and 2, respectively.

Lastly, a 10 consecutive SNP window approach was utilized to characterize regions that have a large effect on the specific trait. The threshold of 1% of additive genetic variance explained is traditionally used to declare important markers [33,34,35] and thus was considered in the present study.

In order to support the better performance of WssGWAS, a genome-wide association analysis was also carried out based on regression of phenotypes on the genotypes of animals for one SNP at a time, using mixed model and score (GRAMMAS) in GenABEL [36] as described by the following general formula:

*y* = μ + ***X**b* + ***S**a* + ***Z**u* + *ε*

where *y* is the vector of trait values; μ is the overall mean; *b* is the vector of fixed effects [crossbreed, sex, contemporary group, farm, abattoir and the covariate of the age at slaughtering]; *a* is the fixed effect of the SNP genotype; *u* and *ε* are vectors of random additive polygenic effects and random residuals, respectively, *u* ∼N(0, ***A***σ^2^_a_) and *ε* ∼N(0, ***I***σ^2^_ε_), where ***A*** is the additive genetic relationship matrix estimated from SNP data using the *ibs* function in GenABEL [36], ***I*** is an identity matrix, and σ^2^_a_ and σ^2^_ε_ are the additive genetic and residual error variances, respectively. ***X***, ***S***, and ***Z*** are the related incidence matrices.

### 2.5. Candidate Genes Identification and Pathway Enrichment Analysis

Based on association mapping outcomes, gene annotations for candidate QTL windows (i.e., over the threshold of 1% of additive genetic variance explained) were obtained using the Biomart platform on Ensembl [18] through the ‘Biomart’ R package and considering the Sscrofa11.1 genome version as the reference map [21].

To obtain a list of possibly overrepresented pathways among the list of positional candidate genes identified, a pathway enrichment analysis was performed using the enrichment function in the PANEV tool [37] which estimates the probability of the overrepresentation for each available pathway on KEGG [38]. Considering the multiple hypothesis testing, the *p*-values were corrected using the Benjamini-Hochberg procedure (FDR) and pathways with FDR less than or equal to 0.05 were considered as significantly enriched.

## 3. Results

### 3.1. Heritability 

Descriptive statistics of the two traits selected for the GWA analysis are provided in Table 1. The traits showed moderate heritability (Table 2), with estimated values of 0.57 ± 0.06 and 0.56 ± 0.08 for UTW and TTW, respectively. This pattern in heritability was expected, as these traits have been used in swine selection for years in Italian dry-cured ham production. Higher heritability values, with higher standard errors, were estimated using traditional ***A*** matrix (Table S4). This result suggested that single-step GBLUP approach provides lower prediction bias compared to traditional ABLUP. A high genetic correlation between the two traits was observed using both ***H*** and ***A*** matrices (0.99 ± 0.008 and 0.99 ± 0.004, respectively).

### 3.2. Genome-Wide Association Mapping

**Figure 1:**
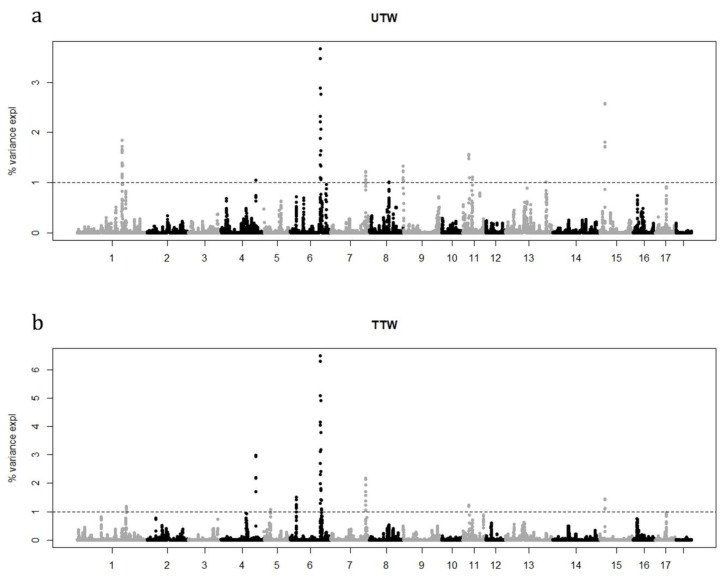
Manhattan plots of the percentage of additive genetic variance explained by windows of 10 adjacent SNPs for UTW, untrimmed thigh weight (**a**) and TTW, trimmed thigh weight (**b**).

A total of 240 pigs were genotyped with the Illumina PorcineSNP60 (~64 K SNPs). After quality control, the final data set consisted of 36,569 SNP for 236 animals. We identified genomic regions associated with the two traits under investigation considering the variance explained by 10 consecutive SNP window estimated by WssGWAS approach. Manhattan plots showing the proportion of genetic variance explained by the 10 SNP window (~0.55 Mb) are in Figure 1. 

A total of nine and eight relevant genomic regions were found to be associated with UTW and TTW traits, respectively. The genomic regions were mainly located on chromosomes 1, 4, 6, 7, 11 and 16 in both traits. Whereas specific QTL signals were detected on chromosome 8, 9, 13 for UTW and chromosome 5 for TTW trait. The most important windows explained the 15.3% and 18.1% of the genetic variance of each trait (Table 3). Same significant windows were observed in both traits on chromosome 1, 4, 7, 11, and 15. A partial overlap was observed for chromosome 6 (~130–134 Mbps), whereas on the same chromosome for TTW trait an exclusive signal was detected around the position ~19.73–20.04 Mbps. A total of 69 and 76 SNPs were highlighted considering the 1% of additive genetic variance threshold. Genes in proximity (≤0.5 Mbps) of the candidate SNPs were identified using the *Sus scrofa* 11.1 reference genome map [22], and a total of 135 and 195 unique positional candidate genes-some of which have been previously reported to be associated with carcass traits in pig and other species-were identified for UTW and TTW, respectively. In total 81 genes resulted in common between the two traits. A summary of each SNP window that explained more than 1% of additive genetic variance and positional candidate genes are presented in Table 4.

Using the single-SNP GWAS approach, significant associations were not detected at Bonferroni-corrected significance level of 0.05 (corresponded to P_nominal value_ < 1.37 × 10^−6^) for both traits. However, considering the complexity of investigated traits and according to other GWAS focusing on similar traits in pig [1,39], since Bonferroni correction acts in stringent manner, several SNPs might be defined as suggestively associated at P_nominal value_ < 5.00 × 10^−5^ and < 5.00 × 10^−4^ thresholds (Figures S1 and S2), as previously reported by other GWAS in livestock [1,39,40,41]. Overall, this result confirmed the effectiveness of WssGWAS approach in our scenario, which is very common in GWA researches for growth and carcass traits in livestock where often a large numbers of individuals have phenotypes and pedigrees but fewer are genotyped.

### 3.3. Pathway Enrichment Analysis

Considering the entire list of unique 249 positional candidate genes, a pathway enrichment analysis was performed using PANEV [37] to identify possible overrepresented pathways. This tool provided a hypergeometric distribution test to calculate significantly enriched biological terms within KEGG ontology database [37]. No pathways resulted were significantly enriched (FDR ≤ 0.05) in our entire gene list nor in the list of candidate genes exclusively detected for UTW and TTW (data not shown).

## 4. Discussion

Over the years, several reports have been focused on dissecting the genetics of pig quantity and quality production traits in many countries [42,43,44,45]. In Italy, the PDO dry-cured ham industry has enormous economic importance (Qualivita-ISMEA, 2019) [2]. Therefore the production of the Italian pig aims essentially to provide thighs able to achieve high technological yield and ideal sensorial characteristics at the end of dry-curing process [3] and several studies have aimed to identify QTL for key traits in PDO ham production [1,8,39,46]. In this context, the prevention of the increase in seasoning loss is clearly of primary importance since it is strictly related to the suitability of meat for salting and its yield at the end of dry-curing process [3]. In this regard, the effect of the increase of thigh weight on the reduction of seasoning loss, as well as on organoleptic quality of hams, has been long recognized [3]. For these reasons, thigh weight still represents an important trait to be considered in selection schemes. In particular, TTW may be of particular interest since many meat defects become visible several hours after death and are particularly noticeable during ham trimming [12]. In this work, we report results of ssGWAS and genetic parameters estimation for UTW and TTW traits in two Italian crossbreeds approved for the dry-cured ham production within the San Daniele PDO circuit. The identification at the genome level of QTLs affecting traits under selection could help to design and monitor selection programs including this information and to support genomic selection strategies that are opening new opportunities in pig breeding for dry-cured ham production [47]. In this regard, traits for association studies need to be developed for a routine recording system collecting parameters that are cheap and simple to be measured but also effective in capturing genetic variability within the population under selection. This latter aspect is a necessary condition for a trait to respond to selection [48] and heritability represents the most important indicator. In our study, a moderate heritability was detected for both UTW and TTW, 0.57 and 0.56 respectively. Our results are within the range of other previous reports [8,49,50] and confirm that these traits can be used as criteria in selection plans to improve dry-cured ham production and ultimately seasoning aptitude. As expected, a very high genetic correlation between the two traits was detected (0.99) indicating that the maximization of thigh yield and carcass conformation has been an achieved goal obtained by selection schemes over the years in Italian heavy pig industry. Furthermore, this result suggests that both traits could be equally considered at slaughtering process. Overall, the same pattern in QTL signals was detected in both traits and this result is consistent with the high genetic correlation detected between the two traits. We found QTL signals on chromosomes 1, 4, 6, 7, 11 and 15 in both traits (Figure 1). Whereas specific QTL signals were detected on chromosomes 8, 9, 13 for UTW and chromosome 5 for TTW trait. In particular, signals on chromosomes 1 and 15 were reported in a recent GWAS for green ham weight [10] and QTL signals on chromosomes 4, 6, 7, and 8 have been also previously described for the same trait [51] or for weight loss at first salting [8]. More in general, signals on chromosomes 4, 6 and 7 are extensively reported in literature associated with growth and carcass traits in pig [52,53,54] and particularly for ham weight trait [1,55,56,57,58,59,60,61,62,63,64]. Focusing on positional candidate gene discovery results, 249 unique genes were identified in proximity (≤0.50 Mbps) of SNP pinpointed within the QTL windows over the 1% of explained genetic variance threshold. In general, the presence of such QTL signals spread across several chromosomes seemed to confirm that muscle development is a complicated physiological process [65]. Although no pathway was statistically significantly enriched, it was observed that many genes were related to muscle fibres formation pathways or already reported in association with skeletal muscle, carcass or feed efficiency traits in pig or other species, notably beef cattle. For example, on chromosome 4, *SLC6A17* (*Solute Carrier Family 6 Member 17*) gene was reported in literature to be correlated with feed/gain ratio in pig [66] whereas *KCNA3* (*Potassium Voltage-Gated Channel Subfamily A Member 3*) gene is known to affect carcass traits in swine [67] and cattle [68]. *PROK1* (*Prokineticin 1*) gene has been reported as candidate gene associated with body weight [69] and *SLC16A4* (*Solute Carrier Family 16 Member 4*) associated with feed efficiency in cattle [70]. The *CSF1* gene (*Colony stimulating factor 1*), which encodes a myokine, is known to be associated with skeletal muscle in human [71] but was also already associated with growth and fatness in the Iberian pig breed [72]. This gene deserves particular mention in the light of its role in PI3K-Akt signaling pathway [73] that is known to be involved during the initial conversion of muscle to meat [74]. *GSTM3* (*Glutathione S-Transferase Mu 3*) was reported as a functional candidate gene in pig with different backfat thicknesses [75]. *AMPD2* (*Adenosine Monophosphate Deaminase 2*) gene is noteworthy since *AMPD* was reported to be a candidate gene for meat production trait in pig and beef cattle [76,77]. Indeed, AMPD is a complex allosteric enzyme encoded by a multigene family in mammals. Previous studies indicated that this gene is involved in energy metabolism and closely related to growth and carcass traits in pig [77]. *SLC16A4* gene was mapped as potential candidate for body length in indigenous cattle breeds [70]. On chromosome 6, *ADGRL2* (*Adhesion G Protein-Coupled Receptor L2*), *CNOT1* (*CCR4-NOT Transcription Complex Subunit 1*) and *SLC38A7* (*Solute Carrier Family 38 Member 7*) were already described as candidate genes for weight [78] and growth [79] traits in Landrace pigs. Whereas *CCDC113* (*Coiled-Coil Domain Containing 113*) and *CFAP20* (*Cilia And Flagella Associated Protein 20*) were recently identified in a genome-wide association study on carcass and meat quality traits in Italian Large White pigs [80]. *KATNB1* (*Katanin Regulatory Subunit B1*) gene was reported as a potential candidate gene for muscle fibre characteristics in pigs [81]. Among the others, *NDRG4* (*NDRG Family Member 4*) and *COQ9* (Coenzyme Q9) genes deserve particular mention. Indeed, *NDRG4* plays a key role in regulating the myogenic differentiation via Akt/CREB activation [82] whereas *COQ9* was reported in association with water holding capacity and with several quality traits in pigs [83]. *PRKACB* (*Protein Kinase CAMP-Activated Catalytic Subunit Beta*) gene is intriguing considering the role played by cAMP-dependent protein kinase in skeletal muscle adaptation [84]. On chromosome 7, *TDP1* (*Tyrosyl-DNA Phosphodiesterase 1*) and *CALM1* (*Calmodulin 1*) genes were recently described as candidate genes associated with ham weight loss at first salting [10]. The effect of *PSMC1* (*Proteasome 26S Subunit*, *ATPase 1*) on growth and carcass traits was also already reported and the use of its polymorphisms in marker-assisted selection for improving beef cattle was suggested [85]. *FOXN3* (*Forkhead Box N3*) gene was proposed as candidate gene affecting intramuscular fat content in swine [86]. *EFCAB11* (*EF-Hand Calcium Binding Domain 11*) gene was recently identified as candidate gene for ham weight at end of salting [10]. On chromosome 15, *MGAT5* (*Alpha-1*,*6-Mannosylglycoprotein 6-Beta-N-Acetylglucosaminyltransferase*) gene is known to be associated with intramuscular fat in pig [87]. This gene was associated with dry matter intake and mid-test metabolic weight in beef cattle [88]. Lastly, it is interesting to note that within the QTL signal on chromosome 1 the *IFT74* (*Intraflagellar Transport 74*) was identified as potential candidate gene, which was already reported in an association study for intramuscular fat content in heavy pigs [89]. 

## 5. Conclusions

Using the ssGBLUP method, moderate heritability values were estimated for UTW and TTW (~0.55) and candidate QTL regions explaining more than 1% of additive genetic variance were identified. In particular, significant QTL signals were highlighted notably on chromosome 1, 4, 6, 7, and 15. A total of 249 unique candidate genes were pinpointed. Notably, some of them were reported in literature in association with growth, carcass, feed efficiency or skeletal muscle development traits in swine or beef cattle. In particular, among the others, *CSF1*, *NDRG4*, *TDP1*, *CALM1*, and *EFCAB11* genes were already described as candidate genes for ham weight traits in pigs. Although, our results should be further verified in independent studies including other pig populations to disentangle the possible effects of specific linkage disequilibrium in our population, overall our findings suggest that UTW and TTW represent interesting traits that deserve to be investigated in future pig breeding programs to improve the dry-cured ham production.

## Figures and Tables

**Table 1:** Descriptive statistics for carcass quality and measured carcass traits on entire population.

Trait * (Units)	Number of Animals with Record	Mean	SD	Min.	Max.
UTW (Kg)	1810	16.69	1.50	11.60	22.70
TTW (Kg)	1202	14.56	1.26	10.55	18.39

* UTW, Untrimmed thigh weight; TTW, Trimmed thigh weight.

**Table 2:** Estimates of heritability (h^2^), additive genetic variance (σ^2^_a_) and phenotypic variance (σ^2^_f_). Standard errors are reported in parenthesis.

Trait *	h^2^		σ^2^_a_		σ^2^_f_	
UTW	0.57	(±0.06)	1.10	(±0.16)	1.91	(±0.09)
TTW	0.56	(±0.08)	0.71	(±0.13)	1.26	(±0.07)

* UTW, Untrimmed thigh weight; TTW, Trimmed thigh weight.

**Table 3:** The top SNP windows that explained the highest proportion of genetic variance of each trait for each chromosome.

Trait *	Chromosome	Average Window Position (bp)	Var (%) **
UTW	1	174,878,065	1.8
	4	109,960,856	1.0
	6	130,886,072	3.7
	7	111,515,733	1.2
	8	85,700,162	1.0
	9	211,402.7	1.3
	11	25,964,018	1.6
	13	180,176,231	1.0
	15	17,747,406	2.6
TTW	1	196,026,548	1.2
	4	109,932,435	3.0
	5	19,850,051	1.1
	6	19,867,435	1.5
	6	131,818,101	6.5
	7	111,483,950	2.2
	11	22,910,267	1.2
	15	17,747,406	1.4

* UTW, Untrimmed thigh weight; TTW, Trimmed thigh weight. ** Percentage of genetic variance explained by window.

**Table 4:** Summary of SNP windows that explained >1% of genetic variance for untrimmed thigh weight, with a list of positional candidate genes for each SNP (≤0.50 Mbps).

Trait *	Chr	QTL Region (Mbps)	SNP	Candidate Genes (≤0.50 Mbps from SNP)
UTW	1	174.09–176.24	rs81349662, rs80948504, rs343652685, rs80782484, rs327453220, rs332492531, rs80819153, rs81271780, rs342853895 rs81236950, rs326407187, rs320846990, rs80818307	*PRPF39*, *MIS18BP1*, *FANCM*, *RPL10L*, *U6*, *SNORD127*, *FKBP3*, *TOGARAM1*, *ENSSSCG00000039829*, *ENSSSCG00000043072*, *ENSSSCG00000043544*, *ENSSSCG00000047027*, *ENSSSCG00000049486*
UTW	4	109.68–110.08	rs80977079, rs80870756, rs80980343, rs81380241	*LRIF1*, *CD53*, *ENSSSCG00000006801*, *KCNA3*, *KCNA10*, *ENSSSCG00000006804*, *PROK1*, *SLC16A4*, *RBM15*, *KCNC4*, *SLC6A17*, *ALX3*, *STRIP1*, *AHCYL1*, *EPS8L3*, *GSTM3*, *GNAT2*, *GNAI3*, *GPR61*, *U6*, *DRAM2*, *ENSSSCG00000028425*, *CEPT1*, *AMPD2*, *ENSSSCG00000033730*, *CSF1*, *KCNA2*, *ENSSSCG00000037808*, *ENSSSCG00000040889*, *ENSSSCG00000041122*, *ENSSSCG00000042722*, *ENSSSCG00000043070*, *ENSSSCG00000043393*, *ENSSSCG00000046426*, *ENSSSCG00000049472*, *ENSSSCG00000051454*, *ENSSSCG00000051784*
UTW	6	130.11–131.38	rs81391496, rs81391505, rs81391507, rs81391515, rs81391518, rs80900111, rs81391526, rs81335828, rs81327100, rs81391487, rs81391501, rs81391472, rs81391555, rs81274576, rs81222864, rs81257397, rs321214830	*TTLL7*, *ADGRL2*, *U6*, *PRKACB*, *ENSSSCG00000044932*, *ENSSSCG00000046121*, *ENSSSCG00000046184*, *ENSSSCG00000048726*, *ENSSSCG00000051159*, *ENSSSCG00000051341*
UTW	7	111.45–111.59	rs80938538, rs80869539, rs80871598, rs80819115, rs80878413	*FOXN3*, *EFCAB11*, *TDP1*, *KCNK13*, *PSMC1*, *NRDE2*, *ENSSSCG00000041236*
UTW	8	85.70–85.70	rs81402068	*INPP4B*, *IL15*, *ZNF330*, *RNF150*, *ENSSSCG00000041112*, *ENSSSCG00000041153*, *ENSSSCG00000041450*, *ENSSSCG00000044609*
UTW	9	0.07–0.38	rs81411123, rs81411485, rs81338651, rs81407864, rs81409222, rs81409931, rs81310106, rs81412401, rs81270995, rs81223860	*TRIM66*, *DENND2B*, *ENSSSCG00000014569*, *NRIP3*, *ENSSSCG00000014575*, *TMEM9B*, *DENND5A*, *TMEM41B*, *ZNF143*, *SNORA23*, *IPO7*, *STK33*, *AKIP1*, *ENSSSCG00000036604*, *SNORA3A*, *ENSSSCG00000041869*, *ENSSSCG00000042439*, *ENSSSCG00000044344*, *ENSSSCG00000044464*, *ENSSSCG00000046669*, *ENSSSCG00000047065*, *ENSSSCG00000049636*, *ENSSSCG00000050234*, *ENSSSCG00000051028*
UTW	11	22.85–35.11	rs81430421, rs80950281, rs81430434, rs80927521, rs80853848, rs81430439, rs81289163, rs81232833	*TSC22D1*, *ENSSSCG00000009425*, *ENSSSCG00000009426*, *ENOX1*, *SMIM2*, *SERP2*, *ENSSSCG00000041790*, *ENSSSCG00000042078*, *ENSSSCG00000042342*, *ENSSSCG00000043875*, *ENSSSCG00000044032*, *ENSSSCG00000046910*, *ENSSSCG00000046928*, *ENSSSCG00000047563*, *ENSSSCG00000049260*, *ENSSSCG00000049729*, *ENSSSCG00000051361*, *ENSSSCG00000051718*
UTW	13	180.18–180.18	rs81284542	*NRIP1*, *USP25*, *ENSSSCG00000042337*, *ENSSSCG00000042532*, *ENSSSCG00000047968*, *ENSSSCG00000051574*, *ENSSSCG00000051785*
UTW	15	17.57–18.00	rs81451598, rs81478999, rs81326202, rs81478982, rs81318409, rs81478797, rs81306466, rs81226590, rs81277838	*ACMSD*, *TMEM163*, *MGAT5*, *ENSSSCG00000040294*, *ENSSSCG00000041827*, *ENSSSCG00000043458*, *ENSSSCG00000045091*, *ENSSSCG00000046082*, *ENSSSCG00000047029*, *ENSSSCG00000048606*, *ENSSSCG00000051576*
TTW	1	195.00–196.54	rs80904604, rs80795061, rs80956668, rs325632167, rs80968730, rs323807748, rs80862783, rs80841106	*GPHB5*, *ENSSSCG00000005121*, *TEK*, *IFT74*, *LRRC19*, *PLAA*, *RHOJ*, *SNORD22*, *PPP2R5E*, *ssc-mir-9832*, *KCNH5*, *U6*, *ENSSSCG00000044869*, *ENSSSCG00000051292*
TTW	4	109.58–110.25	rs80953333, rs80977079, rs80945484, rs80870756, rs80980343, rs80826014, rs80926926, rs80882383, rs81380241, rs80960195	*CHI3L2*, *LRIF1*, *CD53*, *ENSSSCG00000006801*, *KCNA3*, *KCNA10*, *ENSSSCG00000006804*, *PROK1*, *SLC16A4*, *RBM15*, *KCNC4*, *SLC6A17*, *ALX3*, *STRIP1*, *AHCYL1*, *EPS8L3*, *GSTM3*, *GNAT2*, *GNAI3*, *GPR61*, *CYB561D1*, *ATXN7L2*, *SYPL2*, *PSMA5*, *SORT1*, *U6*, *DENND2D*, *DRAM2*, *ENSSSCG00000028425*, *CEPT1*, *AMPD2*, *ENSSSCG00000033730*, *AMIGO1*, *CSF1*, *KCNA2*, *ENSSSCG00000037808m ENSSSCG00000040889*, *ENSSSCG00000041122*, *ENSSSCG00000042722*, *ENSSSCG00000043070*, *ENSSSCG00000043393*, *ENSSSCG00000046426*, *ENSSSCG00000049472*, *ENSSSCG00000050471*, *ENSSSCG00000051040*, *ENSSSCG00000051454*, *ENSSSCG00000051784*
TTW	5	19.82–19.88	rs80818243, rs80800107	*HNRNPA1*, *NFE2*, *COPZ1*, *ENSSSCG00000000291*, *ZNF385A*, *ITGA5*, *NCKAP1L*, *ENSSSCG00000000296*, *PDE1B*, *PPP1R1A*, *TESPA1*, *ENSSSCG00000000312*, *MIR148B*, *U6*, *SMUG1*, *GTSF1*, *ENSSSCG00000033014*, *ENSSSCG00000033458*, *ENSSSCG00000034158*, *NEUROD4*, *ENSSSCG00000036953*, *ENSSSCG00000037462*, *OR10A7*, *CBX5*, *ENSSSCG00000040626*, *ENSSSCG00000041361*, *ENSSSCG00000044790*, *ENSSSCG00000047339*, *ENSSSCG00000048196*, *ENSSSCG00000048965*, *ENSSSCG00000049332*, *ENSSSCG00000050900*, *ENSSSCG00000050921*, *ENSSSCG00000051005*
TTW	6	19.73–20.04	rs81391898, rs81391786, rs334905777, rs81391709, rs81252955, rs81218446, rs81344881	*CNOT1*, *GINS3*, *CCDC113*, *CSNK2A2*, *CFAP20*, *MMP15*, *USB1*, *ZNF319*, *TEPP*, *ENSSSCG00000002811*, *KIFC3*, *KATNB1*, *ADGRG3*, *ADGRG1*, *DRC7*, *PLLP*, *CCL22*, *ENSSSCG00000018402*, *SNORA50A*, *U6*, *NDRG4*, *CCL17*, *ENSSSCG00000024759*, *COQ9*, *POLR2C*, *ADGRG5*, *SETD6*, *PRSS54*, *CCDC102A*, *DOK4*, *CIAPIN1*, *GOT2*, *ENSSSCG00000037660*, *SLC38A7*, *ENSSSCG00000041797*, *ENSSSCG00000043194*, *ENSSSCG00000045991*, *ENSSSCG00000048393*, *ENSSSCG00000051066*
TTW	6	130.11–134.84	rs80930038, rs81391472, rs81391496, rs81391501, rs81391505, rs81391507, rs81327100, rs81274576, rs81335828, rs81222864, rs321214830, rs80900111, rs81391518, rs81391515, rs81257397, rs81391526, rs81391555, rs338651373, rs325952161, rs81391813, rs81278099, rs81340135, rs81347953, rs81306200	*TTLL7*, *ADGRL2*, *ADGRL4*, *IFI44*, *DNAJB4*, *NEXN*, *FUBP1*, *U6*, *IFI44L*, *GIPC2*, *PTGFR*, *PRKACB*, *ENSSSCG00000044932*, *ENSSSCG00000046121*, *ENSSSCG00000046184*, *ENSSSCG00000048726*, *ENSSSCG00000050806*, *ENSSSCG00000051159*, *ENSSSCG00000051341*
TTW	7	111.36–111.66	rs80938538, rs80812481, rs80826832, rs80793518, rs80869539, rs80871598, rs80898146, rs80819115, rs326024106, rs80878413	*FOXN3*, *EFCAB11*, *TDP1*, *KCNK13*, *PSMC1*, *NRDE2*, *CALM1*, *ENSSSCG00000041236*, *ENSSSCG00000046875*, *ENSSSCG00000051310*
TTW	11	22.85–22.97	rs81430421, rs81430434, rs80853848, rs81430439, rs81289163	*TSC22D1*, *ENSSSCG00000009425*, *ENSSSCG00000009426*, *ENOX1*, *SMIM2*, *SERP2*, *ENSSSCG00000041790*, *ENSSSCG00000042078*, *ENSSSCG00000042342*, *ENSSSCG00000044032*, *ENSSSCG00000046910*, *ENSSSCG00000046928*, *ENSSSCG00000047563*, *ENSSSCG00000049260*, *ENSSSCG00000049729*, *ENSSSCG00000051361*
TTW	15	17.57–18.00	rs81451598, rs81478999, rs81326202, rs81478982, rs81318409, rs81478797, rs81306466, rs81226590, rs81277838	*ACMSD*, *TMEM163*, *MGAT5*, *ENSSSCG00000040294*, *ENSSSCG00000041827*, *ENSSSCG00000043458*, *ENSSSCG00000045091 ENSSSCG00000046082*, *ENSSSCG00000047029*, *ENSSSCG00000048606*, *ENSSSCG00000051576*

* UTW, Untrimmed thigh weight; TTW, Trimmed thigh weight.

## Data Availability

The data presented in this study are available on request from the corresponding authors. The data are not publicly available due to confidentiality agreements. Supporting data can be made available to bona fide researchers subject to a non-disclosure agreement.

## Supplementary Materials

The following are available online at https://www.mdpi.com/article/10.3390/ani11061612/s1, Figure S1. Manhattan plots showing the genome-wide associations for UTW (untrimmed tight weight) trait obtained with single-SNP regression approach. Negative log_10_ *p*-values of all SNPs that passed quality control are plotted against their genomic positions. Different chromosomes are distinguished with grey and black colors. The dashed lines indicate the nominal *p*-value of 5.0 × 10^−5^ and 5.0 × 10^−4^, respectively. Figure S2. Manhattan plots showing the genome-wide associations for TTW (trimmed tight weight) trait obtained with single-SNP regression approach. Negative log_10_ *p*-values of all SNPs that passed quality control are plotted against their genomic positions. Different chromosomes are distinguished with grey and black colors. The dashed lines indicate the nominal *p*-value of 5.0 × 10^−5^ and 5.0 × 10^−4^, respectively. Table S1: Formulation, chemical composition and nutritive value of the diets (%, as fed basis) fed to the pigs from 80 to 115 days (Phase A), from 115 to 195 days (Phase B) and from 195 to 270 days Finishing). Table S2: Distribution of SNPs after quality control and average distances on each chromosome. Table S3: Accuracy of genomic breeding values estimated for the three iterations investigated using a five-fold cross validation scheme. Table S4: Heritability (h^2^), additive genetic variance (σ^2^_a_) and phenotypic variance (σ^2^_f_) estimated considering the pedigree-based (***A***) relationship matrix. Standard errors are reported in parenthesis.
