# Supplementary material for: Single-Step Genome Wide Association Study Identifies QTL Signals for Untrimmed and Trimmed Thigh Weight in Italian Crossbred Pigs for Dry-Cured Ham Production
Source: Animals (Basel). 2021 May 29;11(6):1612. doi: 10.3390/ani11061612 (PMC8227816; doi:10.3390/ani11061612)

Figure S2. Manhattan plots showing the genome-wide associations for TTW (trimmed tight weight) trait obtained with single-SNP regression approach. Negative $\log_{10}$ p-values of all SNPs that passed quality control are plotted against their genomic positions. Different chromosomes are distinguished with grey and black colours. The dashed lines indicate the nominal p-value of 5.0E-05 and 5.0E-04, respectively.

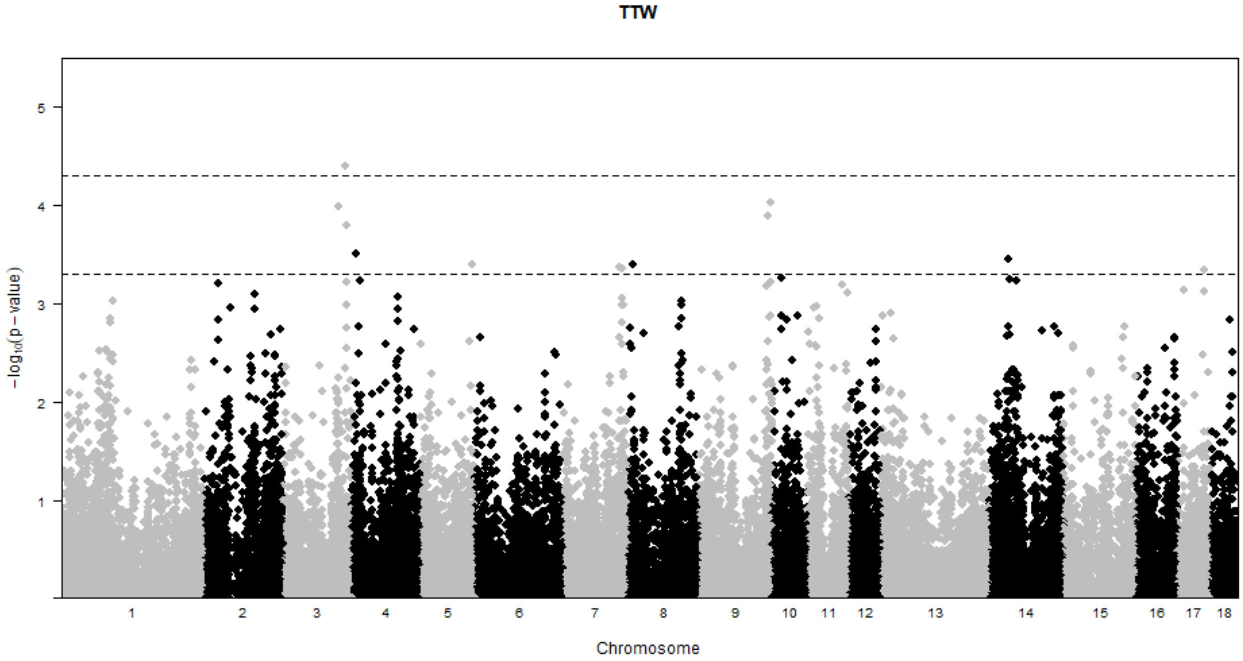

Supplement: Supplementary file 1 [file animals-11-01612-s001.zip › animals-1148087-supplementary/Supplementary Files/Supplementary Figures/Figure S2.pdf]
