# Supplementary material for: Single-Step Genome Wide Association Study Identifies QTL Signals for Untrimmed and Trimmed Thigh Weight in Italian Crossbred Pigs for Dry-Cured Ham Production
Source: Animals (Basel). 2021 May 29;11(6):1612. doi: 10.3390/ani11061612 (PMC8227816; doi:10.3390/ani11061612)
Supplement: Supplementary file 1 [file animals-11-01612-s001.zip › animals-1148087-supplementary/Supplementary Files/Supplementary Tables/Table S1.pdf]

Table S1. Formulation, chemical composition and nutritive value of the diets (%, as fed basis) fed to the pigs from 80 to 115 days (Phase A), from 115 to 195 days (Phase B) and from 195 to 270 days Finishing).

	**Phase A**	**Phase B**	**Finishing**
Ingredients			
Corn	41.68	47.82	47.57
Soybean meal, s.e.	17.82	13.86	10.89
Barley	16.83	16.83	19.8
Bran	8.99	9.39	10.4
Germ flour	3.96	3.47	2.97
Molasses	3.96	3.96	3.96
Ca Carbonate	1.68	1.68	1.68
Palm oil	1.49	1.19	1.19
Beet pulp	1.49		
Dicalcium phosphate	0.5	0.5	0.35
Vitamins Mix	0.5	0.5	0.4
Sodium chloride	0.3	0.3	0.3
Chemical composition			
Dry matter	88.25	87.93	88.52
Concentrate feed	4.58	4	3.75
Crude protein	17.25	15.1	13.35
Ether extracts	4.42	3.95	3.94
Ash	7.16	6.06	5.69
Nitrogen-free extract	66.59	58.81	61.78
Neutral detergent fibre	16.61	14.04	14.63
Lysine	1.67	1.46	1.24
Methionine	0.51	0.47	0.46
Digestible, kcal/kg	3190	3150	3170
