# Supplementary material for: Single-Step Genome Wide Association Study Identifies QTL Signals for Untrimmed and Trimmed Thigh Weight in Italian Crossbred Pigs for Dry-Cured Ham Production
Source: Animals (Basel). 2021 May 29;11(6):1612. doi: 10.3390/ani11061612 (PMC8227816; doi:10.3390/ani11061612)
Supplement: Supplementary file 1 [file animals-11-01612-s001.zip › animals-1148087-supplementary/Supplementary Files/Supplementary Tables/Table S2.pdf]

Table S2. Distribution of SNPs after quality control and average distances on each chromosome.

Chromosome	No. SNPs	Average distance (kb)[a]
1	4147	66.09
2	2460	61.52
3	1947	68.04
4	2560	51.10
5	1567	66.24
6	2396	71.15
7	2311	52.63
8	2012	68.57
9	2012	61.12
10	2278	54.38
11	1358	58.16
12	1175	51.88
13	2817	73.78
14	2811	50.41
15	1969	70.75
16	1340	59.05
17	1220	50.56
18	931	59.41

[a]Average distance between adjacent SNPs on each chromosome
