# Supplementary material for: Single-Step Genome Wide Association Study Identifies QTL Signals for Untrimmed and Trimmed Thigh Weight in Italian Crossbred Pigs for Dry-Cured Ham Production
Source: Animals (Basel). 2021 May 29;11(6):1612. doi: 10.3390/ani11061612 (PMC8227816; doi:10.3390/ani11061612)
Supplement: Supplementary file 1 [file animals-11-01612-s001.zip › animals-1148087-supplementary/Supplementary Files/Supplementary Tables/Table S3.pdf]

Table S3. Accuracy of genomic breeding values estimated for the three iterations investigated using a five-fold cross validation scheme.

Trait	Iteration #	Mean	SD
UTW	1	0.8366	<.000
UTW	2	0.8387	<.000
UTW	3	0.8368	<.000
TTW	1	0.7888	<.000
TTW	2	0.7934	0.001
TTW	3	0.7891	<.000

*UTW, Untrimmed thigh weight; TTW, Trimmed thigh weight.
