# Supplementary material for: Single-Step Genome Wide Association Study Identifies QTL Signals for Untrimmed and Trimmed Thigh Weight in Italian Crossbred Pigs for Dry-Cured Ham Production
Source: Animals (Basel). 2021 May 29;11(6):1612. doi: 10.3390/ani11061612 (PMC8227816; doi:10.3390/ani11061612)
Supplement: Supplementary file 1 [file animals-11-01612-s001.zip › animals-1148087-supplementary/Supplementary Files/Supplementary Tables/Table S4.pdf]

Table S4. Heritability (h^2), additive genetic variance (σ^2_a) and phenotypic variance (σ^2_f) estimated considering the pedigree-based (A) relationship matrix. Standard errors are reported in parenthesis.

**Trait***	**h^2**		**σ^2_a**		**σ^2_f**	
UTW	0.72	(±0.08)	1.75	(±0.26)	2.44	(±0.13)
TTW	0.68	(±0.09)	1.08	(±0.19)	1.58	(±0.10)

*UTW, Untrimmed thigh weight; TTW, Trimmed thigh weight.
